# Fabricating Femtosecond Laser-Induced Periodic Surface Structures with Electrophysical Anisotropy on Amorphous Silicon

**DOI:** 10.3390/nano11010042

**Published:** 2020-12-26

**Authors:** Dmitrii Shuleiko, Mikhail Martyshov, Dmitrii Amasev, Denis Presnov, Stanislav Zabotnov, Leonid Golovan, Andrei Kazanskii, Pavel Kashkarov

**Affiliations:** 1 Faculty of Physics, Lomonosov Moscow State University, 1/2 Leninskie Gory, 119991 Moscow, Russia; martyshov@physics.msu.ru (M.M.); denis.presnov@phys.msu.ru (D.P.); zabotnov@physics.msu.ru (S.Z.); golovan@physics.msu.ru (L.G.); kazanski@phys.msu.ru (A.K.); kashkarov@physics.msu.ru (P.K.); 2 Big Data Storage and Analysis Center, Lomonosov Moscow State University, Lomonosovsky Avenue 27/1, 119192 Moscow, Russia; 3 Prokhorov General Physics Institute of the Russian Academy of Sciences, 38 Vavilova st., 119991 Moscow, Russia; amasev.dmitriy@physics.msu.ru; 4 Skobeltsyn Institute of Nuclear Physics, Lomonosov Moscow State University, 1/2 Leninskie Gory, 119991 Moscow, Russia; 5 Quantum Technology Centre, Lomonosov Moscow State University, 1/35 Leninskie Gory, 119991 Moscow, Russia; 6 National Research Centre “Kurchatov Institute”, 1 Akademika Kurchatova sq., 123182 Moscow, Russia

**Keywords:** laser-induced periodic surface structures, silicon nanocrystals, surface plasmon-polaritons, femtosecond laser pulses, amorphous silicon, electrophysical measurements, Raman spectroscopy

## Abstract

One-dimensional periodic surface structures were formed by femtosecond laser irradiation of amorphous hydrogenated silicon (a-Si:H) films. The a-Si:H laser processing conditions influence on the periodic relief formation as well as correlation of irradiated surfaces structural properties with their electrophysical properties were investigated. The surface structures with the period of 0.88 and 1.12 μm were fabricated at the laser wavelength of 1.25 μm and laser pulse number of 30 and 750, respectively. The orientation of the surface structure is defined by the laser polarization and depends on the concentration of nonequilibrium carriers excited by the femtosecond laser pulses in the near-surface region of the film, which affects a mode of the excited surface electromagnetic wave which is responsible for the periodic relief formation. Femtosecond laser irradiation increases the a-Si:H films conductivity by 3 to 4 orders of magnitude, up to 1.2 × 10^−5^ S∙cm, due to formation of Si nanocrystalline phase with the volume fraction from 17 to 28%. Dark conductivity and photoconductivity anisotropy, observed in the irradiated a-Si:H films is explained by a depolarizing effect inside periodic microscale relief, nonuniform crystalline Si phase distribution, as well as different carrier mobility and lifetime in plane of the studied samples along and perpendicular to the laser-induced periodic surface structures orientation, that was confirmed by the measured photoconductivity and absorption coefficient spectra.

## 1. Introduction

Laser structuring of amorphous hydrogenated silicon (a-Si:H) surfaces has been attracting the attention of scientists for a long time, as it is a promising method for increasing the efficiency of solar cells based on this material. Such processing induces formation of surface inhomogeneities with characteristic dimensions comparable to the visible and near infrared radiation wavelength, which leads to an increase in the optical absorption of the film [1,2,3] due to enhanced scattering of the incident light on the structured surface. Laser modification also induces formation of a crystalline Si phase within a-Si:H films, that sufficiently retards photoelectric properties degradation under the sunlight action to these structures [1,4]. In this case, the irradiated a-Si:H film represents a nanocomposite in the form of silicon nanocrystals embedded into an amorphous matrix [4]. Laser pulses of various duration can be used for a-Si:H films modification, including nanoseconds [5]. However, only irradiation by ultrashort laser pulses enables uniform and accurate structuring of the a-Si:H film, owing to emergence of nonthermal melting mechanism during laser processing [6,7,8].

An additional feature observed during femtosecond laser irradiation of a-Si:H films is the formation of anisotropic laser-induced periodic surface structures (LIPSS or “ripples”). The characteristic scale of such ripples formed in air environment is comparable to the wavelength of the incident radiation [9]. In generally, similar technologies allow fabricating LIPPS with periods, which are significantly smaller than the laser wavelength. For example, femtosecond laser irradiation of semiconductor surfaces in various liquid environments might lead to formation of the LIPSS with subwavelength periods from 70 to 400 nm [10]. It is also possible to produce one-dimensional surface structures with a characteristic transverse size about 10 nm using two femtosecond laser beams with orthogonal polarizations and spatial overlapping [11]. Such structures may be interesting from the point of view of following studying dependencies of their electrical properties on the period. Nonetheless, in the presented work we will pay our attention to classical and easy to produce interference LIPSS with the micron-scale period which is comparable with the wavelength of the used femtosecond radiation.

Formation of such LIPSS is described by a mechanism which consists in interference of surface plasmon-polaritons generated by high-power ultrashort laser pulses with incident light [12,13]. Such LIPSS can serve as a diffraction grating, which is known to increase the efficiency of amorphous/crystalline Si heterojunction solar cells due to localization of incident light at a certain depth of the structure [14]. The formation of one-dimensional surface structures on the a-Si:H film surface also attracts interest for application in polarization optoelectronics, since such modified films demonstrate birefringence, dichroism [15,16], and anisotropy of electrical properties [12,15,17]. In general, for a wider set of materials one of the promising LIPSS applications is polarization-sensitive optical memory fabrication [16] including multilevel and highly stable coding [18]. In such a system, three bits of information or more can be written [19] in a single micron-scaled cell with the LIPSS formed within it. The storage of additional information in the same cell is achieved by variation of the formed LIPSS orientation, as well as dichroism and retardance values of these structures [20]. Finally, femtosecond laser-irradiated thin a-Si:H films containing both silicon nanocrystals and ordered laser-induced surface structures can be used to create hydrophobic coatings, sensors, and thin-film transistors for flat panel displays [9].

It should be noted that a lot of the listed above papers about LIPSS based on a-Si:H [9,15,16,17] have no information about influence of processing conditions on the orientation and period of the formed structures. Though, in the experiments [12] a rotation of the LIPSS direction at the a-Si:H film surface was demonstrated when the number of modifying laser pulses was changed, the observed effect requires theoretical substantiation taking into account the influence of nonequilibrium charge carriers excited in a-Si:H by high-power femtosecond laser pulses. At the same time, laser processing of an a-Si:H film in scanning mode can lead to inhomogeneous crystallization within the film surface plane due the scan lines formation in a case when the laser spot moves continuously in one direction and with a certain step in the orthogonal one [12]. Thus, when analyzing the electrophysical anisotropy of the femtosecond laser-irradiated a-Si:H films, it is necessary to take into account the simultaneous formation of the LIPSS and scan lines, the influence of which must be separated.

Within the above, the aims of our work are the following: to find a correlation between the formation of various LIPSS types and photoexcitation of appropriate surface electromagnetic wave modes taking into account the density of carriers photoinduced in a-Si:H film by femtosecond laser radiation; to analyze a correlation of the modified films structure with their electrical and photoelectrical properties, excluding the possible large-scale scan lines contribution.

## 2. Materials and Methods

Initial a-Si:H films with 600 nm thickness were fabricated by plasma-enhanced chemical vapor deposition [4] on glass substrates. Then the films were irradiated with a laser system Avesta based on a Cr:forsterite crystal by femtosecond laser pulses with the fundamental wavelength *λ* = 1250 nm. The laser pulse duration *τ* = 125 fs and the repetition rate *f* = 10 Hz, respectively. The irradiation was carried out in air at the normal pressure.

**Figure 1:**
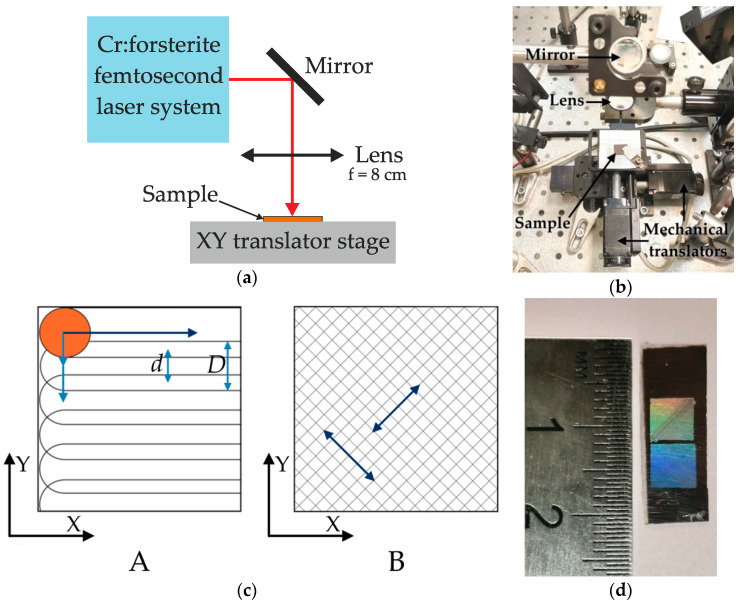
(**a**) Scheme of experimental setup used for processing the a-Si:H films by femtosecond laser pulses. (**b**) Photo of experimental setup. (**c**) Scanning mode templates used during a-Si:H femtosecond laser modification. (**d**) Photo of the areas at a-Si:H film surface, laser-modified in scanning mode B.

The films were processed by femtosecond laser pulses in a scanning mode, which was realized by moving the samples in the horizontal plane (XY) perpendicular to the laser beam. A system of two automated mechanical translators Standa was controlled by a personal computer and used to move the samples. The laser polarization vector was directed along the X axis. The setup scheme and its photo are given in Figure 1a,b, respectively.

Two different scanning modes were used for sample fabrication. The first mode, indicated by the letter A in Figure 1c, was implemented by moving the a-Si:H film in the horizontal plane continuously with a given speed *V* along X axis, and discretely with a given step *d* ≤ *D* along the Y axis, where *D* is the laser spot diameter. As a result, so-called scan lines were formed on the treated area along X axis without unmodified gaps between them. Due to continuous movement along the X axis, the spots on a-Si:H surface from consecutive laser pulses were overlapping, which resulted into a total laser pulses number per the same area *n* = 30 (Table 1).

The second mode, indicated as B in Figure 1c, was realized by simultaneous smooth movement of the a-Si:H film along both axes at the same speed. As a result, scan lines were formed diagonally, at an angle of 45° in relation to the sides of the treated area. At first the entire area of the sample was scanned along the diagonal lying in the 1 and 3 quadrants of the coordinate plane, and then the same area was scanned along the diagonal lying in the 2 and 4 quadrants. However, in this case the scanning was performed at lower speed *V* which resulted into a high number of the laser pulses *n* per the same area of the sample. In this case better uniformity of the treated surface was achieved in comparison with the mode A, and a possible contribution to the conductivity anisotropy related to scan lines presence was excluded.

Several square 4.5 × 4.5 mm^2^ areas at the surfaces of a-Si:H films were irradiated with each template to provide enough treated surface for scanning electron microscopy (SEM) investigation as well as the electrophysical measurements. An example of irradiated areas at a-Si:H film surface is given in Figure 1d. The areas obtained using the scanning mode A were indicated as sample 1, while the areas irradiated by scanning mode B as sample 2, according to Table 1.

Images of the modified surface of the films were obtained by scanning electron microscopy (SEM) using a Carl Zeiss Supra 40 microscope. The phase composition analysis of both initial a-Si:H film and irradiated samples was carried out by Raman spectroscopy using a Horiba JobinYvon HR800 Raman microscope with excitation by He-Ne laser at the wavelength of 633 nm.

The electrical properties of initial and femtosecond laser-irradiated a-Si:H films were studied using a Keithly 6487 picoammeter and an optical nitrogen cryostat in vacuum of 10^−3^ Pa. The electrical measurements were carried out at direct current applied in the directions which are coplanar to the film surface. For this purpose, each time 4 square aluminum electric contacts were deposited by thermal resistive sputtering at the surface of unmodified a-Si:H and each modified area. The electric contact edges were directed both along and orthogonal to the fabricated LIPPS. Thus, the electrical measurements were conducted in two mutually orthogonal directions in surface plane.

To remove water from the surface, all samples were annealed at temperature of 433 K (~160 °C) in vacuum for 5 min before the electrical measurements. After that dark conductivity temperature dependencies were measured while the samples were cooling down to 273 K with a step of 2 K. For photoelectrical measurements, the samples were illuminated in the visible and near-infrared spectral range from 1 to 2.2 eV using a halogen incandescent lamp and a Lot Oriel monochromator. Absorption coefficient spectra of both initial a-Si:H film and irradiated samples were measured using a constant photocurrent method (CPM).

## 3. Theoretical Modeling of the LIPSS Formation

The formation of a periodic relief on metal, dielectric, and semiconductor surfaces irradiated by femtosecond laser pulses is often associated with the surface electromagnetic waves (SEW) generation [21]. For the normal LIPSS formation, the conditions for a certain SEW mode excitation—a surface plasmon-polariton—must be satisfied at the interface between two media [13]:

(1) Reε2<0, |Reε2|>Reε1>0,

where *ε*_1_ = 1 is the dielectric permittivity corresponding to air, and ε_2_ corresponds to the irradiated material. Therefore, in the case of semiconductors and dielectrics, including a-Si:H, it is necessary to achieve the negative sign of Re *ε*_2_ for surface plasmon-polariton excitation [12,13,21]. The conditions (1) can arise in a-Si:H under irradiation with high-power femtosecond laser pulses. Due to intense photoexcitation by incident radiation a high concentration of nonequilibrium charge carriers is generated, which leads to metallization of irradiated surface. According to the Drude model, relations (1) are satisfied when the nonequilibrium charge carriers density reaches the threshold value *N*_0_, which corresponds to the Drude plasma resonance [13]:

(2) $$N_{0}=\left(\omega^{2}+\gamma^{2}\right)\left(\varepsilon +1\right)m^{*}/4\pi e_{0}^{2},$$

where *ω* is the incident laser radiation frequency, *γ* = *e*_0_/(m*μ) is the collision frequency, of the material, m* and *e*_0_ are the effective mass and charge of an electron, respectively, *ε* is the static dielectric permittivity for non-excited a-Si:H and *μ* is the electron mobility for the irradiated material.

On the other hand, the maximum value of nonequilibrium charge carrier’s density *N* achieved in the a-Si:H film upon femtosecond laser irradiation can be estimated by the differential equation:

(3) $$\frac{dN}{dt}=\frac{\left(1-R\right)I\left(t\right)}{\hbar \omega }\alpha +\frac{{\left(1-R\right)}^{2}I^{2}\left(t\right)}{2\hbar \omega }\beta ,$$

where *α*, *β* are the one-photon and two-photon absorption coefficients, respectively; *R* is the reflection coefficient and *I*(*t*) is the incident laser radiation intensity. Temporal distribution of the laser radiation intensity within the pulse for the Equation (3) was described as follows [22]:

(4) $$I(t)=I_{0}\;t\exp(-4t/\tau ),\;\int_{0}^{\infty }I\left(t\right)dt=Q$$

where *Q* = 0.5 J/cm^2^ is the fluence of a laser pulse.

The following values of the absorption coefficients for a-Si:H were used in the Equation (3): *α* = 10 cm^−1^ [23], *β* = 37 cm/GW [24]. The reflection coefficient *R* is given by the formula:

(5) $$R={\left|\frac{\sqrt{\tilde{\varepsilon }}-1}{\sqrt{\tilde{\varepsilon }}+1}\right|}^{2},$$

where $\tilde{\varepsilon }$ is the complex dielectric permittivity of irradiated material, which also depends on *N*, according to the Drude model [13].

In order to analyze the formation of LIPSS with different periods and orientations, depending on the nonequilibrium charge carriers concentration in the irradiated material, the model proposed by J Sipe et al. [25] and modified by J. Bonse et al. [26,27,28] was used. This so-called Sipe-Drude model utilizes so-called efficacy factor *η*(*κ_x_*, *κ_y_*), which indicates the probability of LIPSS formation with period *Λ* under the action of incident laser radiation and in the presence of SEW excitation. The values of *κ_x_* and *κ_y_* are the surface lattice wave vector ***κ*** components normalized by the incident laser radiation wavelength:

|**κ**| = λ/Λ (6)

The model allows for relating the density of nonequilibrium charge carriers *N* excited by a laser pulse, the dielectric permittivity of the irradiated material, and the period of the formed LIPSS. The laser radiation parameters, such as wavelength, polarization, and angle of incidence, as well as the roughness of the surface are also taken into account.

The numerical calculation of *η*(*κ_x_*, *κ_y_*), was carried out using the system of equations for the s-polarized radiation with a wavelength of 1.25 μm at normal incidence (*Θ* = 0) [27]. The shape and filling factors determining the surface roughness were taken equal to 0.4 and 0.1, respectively, in accordance with [25]. The direction of the polarization vector was taken parallel to the *x* axis.

## 4. Experimental Results and Discussion

### 4.1. LIPSS Formation on the a-Si:H Film Surface

**Figure 2:**
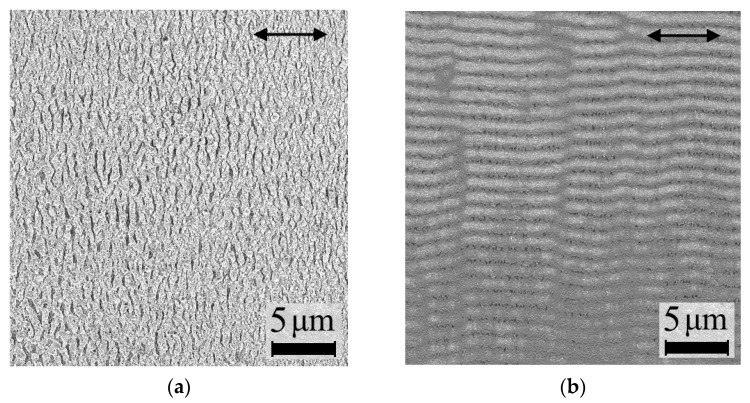

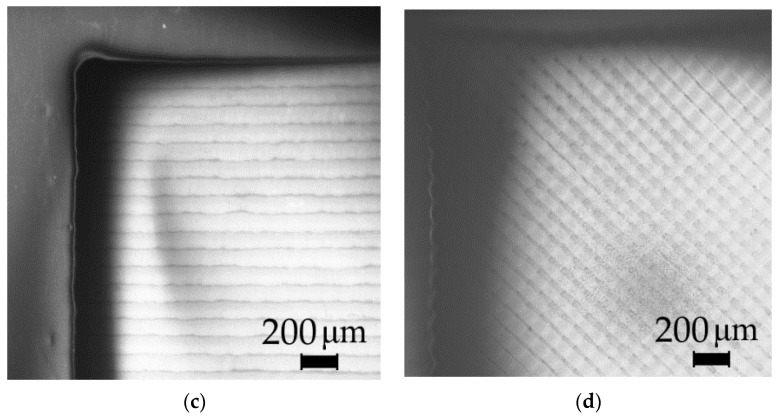
Images of femtosecond laser-modified surfaces of (**a**) sample 1 and (**b**) sample 2, obtained by SEM at 5000× magnification. The arrows indicate the polarization of the femtosecond laser pulses. SEM images of femtosecond laser-modified surfaces of (**c**) sample 1 and (**d**) sample 2, obtained at 70× magnification.

By means of SEM various one-dimensional periodic structures on the surface of the irradiated a-Si:H films were revealed. The orientation of these LIPSS changes depending on the number of femtosecond laser pulses *n*. Formation of surface gratings with ridges oriented orthogonally to the polarization vector of the modifying laser radiation was observed for the sample 1 at *n* = 30. The corresponding wave vector ***κ*** of these structures is parallel to the laser polarization. Such so-called “normal LIPSS” [25] are presented in Figure 2a. The period *Λ* of the lattice is 0.88 ± 0.03 µm.

At the surface of sample 2, modified with higher number of laser pulses *n* = 750, the formation of LIPSS with ridges orientation along the laser polarization is observed (Figure 2b). Such one-dimensional structures are called “anomalous LIPSS” [9], their wave vector ***κ*** is directed orthogonally to the laser polarization, and their period is *Λ =* 1.12 ± 0.02 μm. Additionally, the scan lines with the width *d* = 150 μm are formed during irradiation of sample 1 as can be seen in Figure 2c. In this case the laser spot diameter is *D* = 200 μm, thus no unmodified gaps between the scan lines are formed. It also can be seen in Figure 2d that for the sample 2 the scan lines are directed at 45° to the edges of the modified area.

It should be noted that the LIPSS of both types have periods close to the wavelength *λ* of used femtosecond laser pulses, but smaller than *λ*. The decrease of the LIPSS period compared to incident laser radiation varies from few percent for the second structure type and up to 30% for the first structure. Additionally, the orientation of all types of the structures is determined only by the direction of the femtosecond laser pulses polarization vector and does not depend on the laser beam scanning direction.

### 4.2. Raman Spectra Analysis for Irradiated and Unirradiated a-Si:H Films

**Figure 3:**
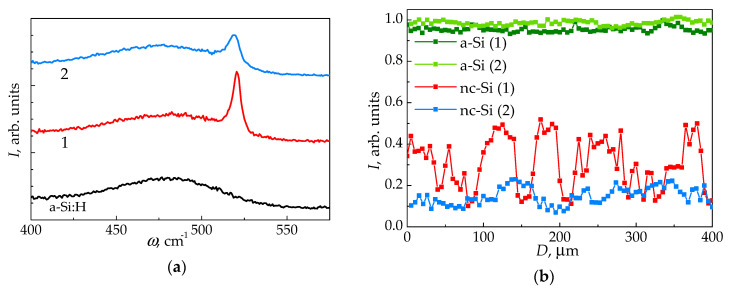
(**a**) Raman spectra for unirradiated amorphous silicon, as well as for samples 1 and 2 normalized by maximum intensity at ω_A_. For sample 1, the spectrum corresponds to the middle of the scan line. (**b**) Integrated TO phonon modes intensities corresponding to a-Si and nc-Si for the samples 1 and 2, mapped in the direction orthogonal to the scan lines, and normalized by the maximum integrated a-Si:H TO phonon mode intensity.

To determine the phase composition of the films before the electrical measurements, the Raman spectra were obtained for both initial a-Si:H and irradiated samples. The presence of a wide band with a maximum at *ω*_A_ = 480 cm^−1^, which is characteristic to amorphous silicon, can be seen in Raman spectra of all the samples in Figure 3a. Along with it, a narrow line near *ω*_C_ = 520 cm^−1^, which corresponds to crystalline (nanocrystalline) silicon (nc-Si) [4,29], is present only in the irradiated samples.

The crystalline silicon (c-Si) phase volume fraction *f*_C_ in the irradiated films was calculated using the following expression [29]:

(7) $$f_{C}=\frac{I_{C}}{\sigma_{0}I_{A}+I_{C}},$$

where *I*_A_ and *I*_C_ are the integrated intensities of TO phonon modes corresponding to the frequencies *ω*_A_ and *ω*_C_, *σ*_0_ is the empirical ratio of the Raman scattering integrated cross sections for the crystalline and amorphous silicon phases, which is determined by the formed silicon nanocrystals size [30]. The size of nanocrystals estimated from the shift of the Raman line at 520 cm^−1^ [30] is ≈3.6 nm for sample 2 and ≈5 nm for sample 1. Corresponding σ_0_ values varied from 0.9 to 0.6, while for bulk c-Si this value equals 0.1 [30]. Obtained according to (7) values of the nc-Si phase volume fraction for sample 1 and 2 are 28 ± 16% and 17 ± 4%, respectively. As can be seen in Figure 3b, the nc-Si phase distribution is significantly less homogeneous within the sample 1, compared to sample 2. This can be explained by the difference in scan lines formation on the irradiated surface when using different scanning modes. As a result, fluence is more nonuniformly distributed within the treated area cross section at applying the one-pass scanning mode A than in case of multi-pass mode B (Figure 1c).

The estimation of volume fractions of the c-Si phase within the samples is important for given below electrical study as well as calculations of the nonequilibrium electrons concentration generated into the conduction band during LIPSS formation; as such, simulation requires the values of the electron mobility and effective mass for the irradiated material.

### 4.3. Modeling of the LIPSS Formation on a-Si:H Surfaces

The formation of LIPSS with a period close to the incident radiation wavelength on the a-Si:H film requires the excitation of a lot of nonequilibrium electrons into the conduction band, within the surface layer of the film. According to expression (2), in case of a-Si:H surface irradiation by femtosecond laser pulses with 1.25 μm wavelength, the threshold value of the nonequilibrium carriers density required for plasmon-polariton generation is *N*_0_ = 8.2∙10^21^ cm^−3^. The dielectric permittivity value *ε* = 9.7 was used in calculations, according to [12]. It should be noted that in the irradiated samples the formation of nc-Si phase was observed, as was shown in Section 4.2. Therefore, the value of the electron mobility *μ* for modified a-Si:H films was taken equal to 30 cm^2^/V∙s, which corresponds to microcrystalline silicon, according to Bergren et al. [31]. Note that the *μ* value used in calculation is almost an order of magnitude higher than the charge carrier mobility in a-Si:H (≈4.5 cm^2^/V∙s), but smaller than carrier mobility *μ* for bulk c-Si ≈ 1300 cm^2^/V∙s [32].

The maximum value of the nonequilibrium charge carriers density *N* is estimated from the Equation (3) as 1.2∙10^22^ cm^−3^ at the laser pulse fluence *Q* = 0.5 J/cm^2^ and duration *τ* = 125 fs. The obtained value for *N* is higher than the threshold value *N*_0_. Thus, femtosecond laser pulses used in the experiment allow for achieving of high carrier density within the surface layer of a-Si:H film, that is necessary for LIPSS formation.

**Figure 4:**
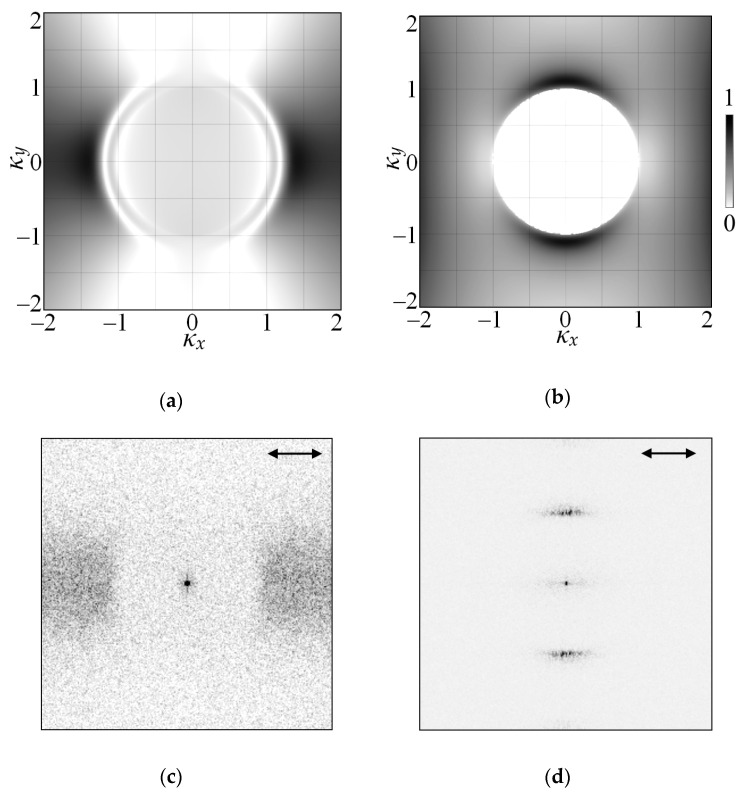
Results of the efficacy factor *η*(*κ_x_*, *κ_y_*) modeling for the samples (**a**) 1 and (**b**) 2, and their comparison with the Fourier-transformed SEM images of the LIPSS experimentally observed on the surface of corresponding samples (**c**,**d**). The arrows indicate the orientation of the laser radiation polarization vector.

The LIPSS modeling according to the Sipe-Drude theory [25,26,27,28] was conducted by varying the *N* values until corresponding *η*(*κ_x_*, *κ_y_*) two-dimension distributions (Figure 4a,b) match Fourier transform images (Figure 4c,d) of the real ripples shown in Figure 2a,b. As a result, the nonequilibrium electrons concentrations *N* and complex dielectric permittivities *ε*, which correspond to the observed ripples formation conditions, were determined. Obtained *N* and *ε* values are given in the Table 2.

As can be seen, the nonequilibrium electrons concentration exceeds the threshold value *N*_0_ for the normal LIPSS, which corresponds to plasmon-polariton excitation conditions. On the other hand, for the sample 2 containing anomalous ripples, which are parallel to the laser polarization, the obtained *N* < *N*_0_. This result is consistent with the mechanism of anomalous ripples formation proposed in [12,13].

According to this mechanism, the decrease of the nonequilibrium electrons concentration in near-surface layer of the film is caused by growth of electron thermal emission at increase of the laser pulses number. This effect is associated with intensified heating of the a-Si:H film surface due to the presence of a periodic surface relief formed by previous laser pulses [12]. Namely, a positive feedback emerges between the surface relief formation by femtosecond laser pulses and an increase in the absorption coefficient of the film in course of time. The increased optical absorption of the film is associated with more efficient scattering of incident radiation by the surface inhomogeneities, formed by previous laser pulses. Such inhomogenities enhance scattering of the incident light with wavelength close to their characteristic dimensions [1] according to Mie theory framework. In our case the characteristic dimension of scatterers is set by LIPSS period, which is close to the wavelength of the laser radiation used. Further, the decreased concentration of nonequilibrium electrons, generated by the laser pulse, affects the sign of the dielectric permittivity real part Re *ε*. As can be seen in Table 2, the value of Re *ε*, corresponding to the obtained *N* values, is negative for the normal LIPSS that are directed orthogonally to the femtosecond laser pulses polarization. Contrary, in case of anomalous LIPSS formation, when the ripples are directed along the polarization of incident femtosecond laser pulses, corresponding Re *ε*, is positive [13]. Such alteration of the Re ε sign in the near-surface region of the film from negative to positive enables excitation of a different SEW mode (TE instead of TM), and consequently the direction of the LIPSS changes. Thus, the period and orientation of both LIPSS types observed in the experiment are in a good agreement with the obtained values for the complex dielectric permittivity *ε* and the listed above mechanisms of their fabrication.

In addition, it should be noted that the periods of both normal and anomalous surface lattices formed by femtosecond laser pulses at the a-Si:H surface in the present work are smaller than those obtained by our group in the previous experiments using a lower laser fluence [12,21]. According to modeling within Sipe theory such decrease of the LIPSS period is attributed to a lower nonequilibrium electrons density achieved during laser processing. An explanation of the observed effect may be similarly given by increased intensity of the thermal emission of electrons from the surface of the a-Si:H due to a stronger heating of the film surface when femtosecond pulses with a higher fluence are applied [33,34]. An additional effect on a decrease in the period of the formed LIPSS can be provided by an increase in the contrast of the relief formed by laser pulses with a higher fluence. According to [35], as the depth of the ablated grooves of the LIPSS increases, the resonant wavelength of the surface electromagnetic wave shifts to smaller values.

### 4.4. Electrical and Photoelectrical Properties of the Modified a-Si:H Surface

**Figure 5:**
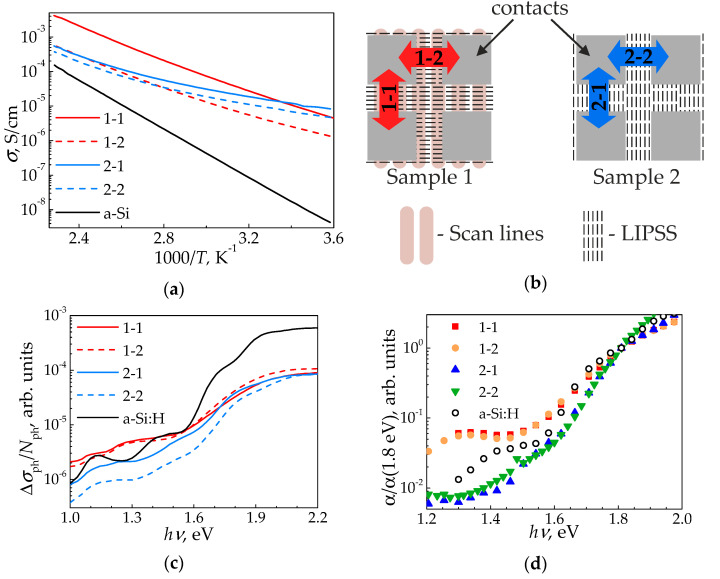
(**a**) Temperature dependencies of dark conductivity *σ*. (**b**) Electric contacts geometry relative to scan lines and LIPSS. Directions of the electric field vector ***E*** are marked as 1-1, 1-2, 2-1, and 2-2. (**c**) Spectra of photoconductivity *σ*_ph_, normalized on the number of photons *N*_ph_. (**d**) Spectra of absorption coefficient *α*, normalized on its value at the mobility gap with for a-Si:H (1.8 eV).

The dark conductivity temperature dependencies of the unmodified a-Si:H and samples 1, 2 are given in Figure 5a. Each curve corresponds to different applied electric field vectors ***E*** relative to scan lines and formed LIPSS, according to Figure 5b. It is worth to mention that the scheme for sample 2 in Figure 5b do not show the scan lines, since they are directed at ±45° angles to the applied ***E*** accordingly the scanning mode B (Figure 1c), and therefore do not affect the conductivity.

As can be seen from Figure 5a, the dark conductivity curves for all the irradiated samples lie higher and have a lesser slope than for the initial a-Si:H, which is explained by the nc-Si phase contribution to the conductivity of these samples. Additionally, the slope of conductivity temperature dependencies *σ*(*T*) for all the irradiated samples demonstrate increase with temperature growth (corresponds to 1/T abscissa decrease in Figure 5a). It indicates an increase of the activation energy, which can be calculated from the temperature curve slope.

Since, in contrast to the initial a-Si:H, the modified regions of the film had a nonmonotonic dark conductivity temperature dependencies, the activation energies were calculated by approximating the obtained experimental curves by an exponent in a certain temperature ranges. In this case, the dark conductivity temperature dependence is described as follows:

(8) $$\sigma =\sigma_{0}exp\left(-\frac{E_{A}}{kT}\right),$$

where *σ*_0_ is a constant, *E_A_* is the activation energy, *T* is the temperature, *k* is the Boltzmann constant. To calculate the activation energy at temperatures of 300 and 400 K, the approximation temperature ranges from 273 to 333 K and from 373 to 433 K were taken, respectively. The values obtained in two mutually orthogonal directions for each sample were the same, taking into account the error of up to 0.05 eV. Obtained activation energies vary from 0.35 to 0.5 eV for sample 1, and from 0.21 eV to 0.47 eV for sample 2 with increasing the temperature from 300 to 400 K. Contrary, the activation energy of the initial a-Si:H is constant and equals 0.7 eV. The activation energy increase can be explained by simultaneous coexistence of both amorphous and nc-Si phases in the modified samples, when the amorphous phase contributes noticeably to dark conductivity only at higher temperatures.

The electrical measurements revealed that specific dark conductivity of a-Si:H films increased by 2 to 3 orders of magnitude as a result of femtosecond laser treatment (Table 3). On the other hand, the photoconductivity, calculated as the difference between the conductivity of illuminated sample and the dark conductivity, decreased by 4 times and more for irradiated samples. It should be clarified that observed dark conductivity increase is mainly influenced by the formation of more conductive nc-Si phase within a-Si:H under the action of high-power femtosecond laser pulses, which was confirmed by Raman spectroscopy in Section 4.2. Despite the nc-Si phase volume fraction that is almost 2 times smaller in sample 2 than in sample 1, as was calculated above, the dark conductivities of both samples were close. Such result can be explained by a more uniform nc-Si phase distribution within the sample 2, processed according to the template B, compared to the sample 1, modified using the template A with the scan lines formation. In the latter case, using template A leads to formation of the regions with significantly reduced nc-Si phase volume fraction *f*_C_, within the sample along the scan lines edges, as can be seen in Figure 3b. In these regions, the *f*_C_ value can be lower than the percolation threshold for nc-Si, which for a homogeneous and isotropic system of silicon nanocrystals randomly distributed in an amorphous Si matrix corresponds to *f*_C_ = 16% [36]. Thus, the conductivity of such regions within the modified film will be determined mainly by the poorly conducting amorphous phase, which leads to reduction of the resulting dark conductivity of the sample 1, especially in the direction which is perpendicular to the scan lines.

Additionally, in-plane anisotropy of conductivity was revealed for the irradiated samples. The dark conductivity values differ up to 4 times in mutually orthogonal directions for sample 1 and up to 1.8 times–for sample 2, as shown in Table 3, while the dark conductivity of initial a-Si:H film is isotropic.

The dark conductivity anisotropy observed for both irradiated samples might be caused both by the form anisotropy of LIPSS [37] and the nonuniform nc-Si phase distribution within scan lines [12]. However, according to Figure 5b and Table 3 the dark conductivity is higher along the scan lines direction (geometry 1-1) than along the LIPSS (geometry 1-2) at room temperature. Thus, the scan lines presence has a stronger effect on the dark conductivity anisotropy. Contrarily, for the sample 2 the dark conductivity is higher along the LIPSS direction. In this case the scan lines were formed at the same angle of 45° to all the electric contacts edges, according to the template B in Figure 1c, and their influence on conductivity was minimized. In other words, conductivity anisotropy of the sample 2 is explained by the pronounced orientation-dependent depolarization of an external electric field inside the micron-scale LIPSS [12].

An additional contribution to the observed effect may as well be caused by anisotropic crystalline and amorphous Si phase distribution within LIPSS, according to [9,38]. In presence of the LIPSS with high amplitude on the surface of a-Si:H film, the charge transport along the LIPSS direction occurs in the highly conductive nc-Si phase which is mainly distributed along the LIPSS ridges [38]. At the same time, in the orthogonal direction, the charge transport paths are crossed by the ablated valleys of the surface relief, which contain mainly an amorphous phase with significantly lower conductivity than the nc-Si. In this case the magnitude of the conductivity anisotropy can be influenced by the depth of the surface relief. However, the period of such relief should not affect the conductivity: though the number of the ablated valleys per unit area is higher in case of small period of LIPSS, the amount of nc-Si phase in the single LIPSS ridge is also proportionally smaller.

It is worth to note that the electrical properties of the a-Si:H films modified by femtosecond laser pulses in air, including the conductivity anisotropy, can be affected by the surface oxidation, which was demonstrated in several works [9,17]. However, strong oxidation is accompanied by a change in a Raman spectrum, when the wide band at 480 cm^−1^ corresponding to a-Si:H broadens [39] or has significantly reduced intensity [17,40]. Such oxidized a-Si:H films can demonstrate extremely low conductivity, even despite the large c-Si volume fraction of 70% [17]. Additionally, Si surfaces passivized by a SiO_2_ layer of 15 nm thickness demonstrate nonlinear conductivity [41]. However, in our case, no similar changes in the Raman spectra (Figure 3a), as well as in the conductivity dependencies (Figure 5a,c), were observed. It indicates that the probable silicon oxide phase formation does not significantly affect the conductivity of irradiated a-Si:H films.

Figure 5c as well as Table 3 also demonstrates pronounced anisotropy of the photoconductivity for sample 2. It might be related either to anisotropy of the electron and hole (marked as n and p in subscript indices, respectively) lifetime *τ*_n,p_ and mobility *µ*_n,p_, or by the features of the film absorption coefficient *α*:

Δ*σ*_ph_ = *e*_0_(*µ*_n_*τ*_n_ + *µ*_p_*τ*_p_)G(*α*), (9)

where *G*(*α*) is the charge carrier generation rate. However, the spectral dependencies of the absorption coefficient α given in Figure 5d demonstrate isotropic behavior for sample 2. Thus, observed photoconductivity anisotropy is mostly affected by the *τ*_n,p_ and *µ*_n,p_ anisotropy in relation to the directions along and orthogonal to the LIPSS. This anisotropy may be caused by the different a-Si and nc-Si phase distributions inside the LIPSS. In this consideration along the LIPSS the carriers transport occurs mainly through nc-Si phase, due to its appropriate distribution within the surface relief according to [9], while in the orthogonal direction there is an alternation of a-Si and nc-Si phases. At the same time, the photoconductivity of sample 1 is almost isotropic (Figure 5c), considering the error; the photoconductivity of unirradiated a-Si:H is also isotropic.

In the 1.1–1.5 eV spectral range the photoconductivity spectral dependencies for sample 1 lie higher or near, than one for the initial a-Si:H, while for sample 2 these dependencies are lower, as can be seen in Figure 5c. This indicates a high optical absorption of sample 1 in this range, and low–for sample 2 compared to the initial film, which is confirmed by the absorption spectra obtained by CPM and given in Figure 5d. On the other hand, the photoconductivity above 1.6 eV spectral range for both irradiated samples are smaller compared to a-Si:H one.

In general, the spectral dependence of absorption coefficient is determined by the concentration of defects within the mobility gap, such as dangling bonds. In the 1.1–1.5 eV range the higher optical absorption of sample 1 compared to the initial film, indicates an increase of dangling bonds concentration within the sample 1, which may be caused by femtosecond laser-induced dehydrogenation of a-Si:H. The absorption spectrum in the range of 1.5–1.7 eV also allows to determine the disordering degree of the material. Optical transitions in this range correspond to the absorption edge and are described by the dependence:

*α*(*hν*) = *α*_0_exp(−*hν*/*E*_U_), (10)

where *E*_U_ is the Urbach energy, which describes the band tails width for the disordered material and *α*_0_ is an absorption normalization constant, which depends on the material structure. Approximated from Figure 5d according to Equation (10) *E*_U_ = 65 ± 4 and 62 ± 6 eV for the unmodified film and sample 2, respectively: however for sample 1 *E*_U_ = 100 ± 4 eV. Obtained value is almost twice higher than for the initial a-Si:H, indicating wider band tails and, therefore, greater lattice disorder within the sample 1. Thus, the increased absorption of sample 1 can be explained by the nonuniform femtosecond laser-induced crystallization using the scanning mode A that is accompanied by effective dehydrogenation and dangling bonds formation.

Optical absorption of a femtosecond laser-irradiated a-Si:H film in the 1.1–1.5 eV range can be additionally increased compared to initial a-Si:H due to formation of surface relief with the typical subwavelength dimensions (the periods) according to Mie theory [1], as was mentioned in Section 4.3.

Decreased optical absorption in the 1.1–1.5 eV range for the sample 2, compared to unmodified a-Si:H and sample 1, most likely, can be attributed to excessive material ablation at *n* = 750 pulses from surface in comparison to sample 1 fabricated at 25 times less exposure of *n* = 30 pulses. As a result, the film thickness decreases, and the number of absorbed photons in the smaller volume becomes lower too. Decreased photoconductivity above 1.6 eV spectral range for both irradiated samples compared to a-Si:H can be attributed to a decrease of a-Si:H phase volume fraction due to laser-induced crystallization while optical absorption of c-Si in this spectral diapason is lower compared to a-Si:H [42,43].

## 5. Conclusions

In summary, the possibility of the subwavelength periodical relief formation at a-Si:H surface by femtosecond laser irradiation in different scanning modes by the laser beam was demonstrated. According to theoretical simulation the orientation of the formed surface structures depends on the density of nonequilibrium electrons photoexcited into conduction band by the femtosecond laser pulse during processing. The increase in the number of laser pulses leads to a stronger heating of the a-Si:H film owing to optical absorption enhance by the LIPSS which formed on previous irradiation steps. The heating, in turn, induces thermal emission of electrons from the irradiated surface and leads to change the dielectric permittivity value in the near surface layer during irradiation step by step. As a result, the photoexcited SEW changes its type from TM to TE and, as a consequence, the direction of the formed surface lattice is changed.

The formation of nc-Si phase with a volume fraction from 17 to 28% within the modified a-Si:H films increased their dark conductivity by 2 to 3 orders of magnitude compared to the nonirradiated film. Simultaneously observed pronounced electrophysical anisotropy of femtosecond laser-irradiated films can be attributed both to scan lines formation and LIPSS existence. The dark conductivity values for two orthogonal directions in surface plane may differ up to 4 times due to LIPSS form anisotropy and the nonuniform nc-Si phase distribution within the scan lines. The photoconductivity of the samples after irradiation decreased by 4 times and more due to amorphous silicon phase volume fraction reduction caused by material ablation and crystallization. Furthermore, the anisotropy of the photoconductivity is reached and explained by different charge carrier lifetime and mobility along and perpendicular to the LIPSS direction that is confirmed by the measured photoconductivity and absorption coefficient spectra.

## Figures and Tables

**Table 1:** Sample processing parameters.

Sample	Scanning Mode (Figure 1c)	Laser Pulse Fluence *Q* (J/cm^2^)	Laser Spot Diameter *D* (μm)	Number of Laser Pulses *n*
1	A	0.5	150	30
2	B			750

**Table 2:** LIPSS period and orientation, nonequilibrium electrons concentration *N* and the dielectric permittivity ε at the surface of a-Si:H films during irradiation with femtosecond laser pulses.

Sample	LIPSS Period (μm)	LIPSS Direction Relative to Polarization	*N* (cm^−3^)	*ε*
1	0.88 ± 0.03	⊥	9.3∙× 10^21^	−2.3 + 0.4*i*
2	1.12 ± 0.02	∥	6.5 × 10^21^	1.3 + 0.3*i*

**Table 3:** Dark conductivity and photoconductivity for the samples 1, 2 for different directions of the applied electric field **E** relative to LIPSS and scan lines, and for the unmodified a-Si:H film. The data is obtained at room temperature (293 K).

Sample	Direction of E	Dark Conductivity (10^−6^ S/cm)	Photoconductivity (10^−6^ S/cm)
1	⊥ LIPSS, ‖ scan lines	11 ± 2	0.8 ± 0.2
	‖ LIPSS, ⊥ scan lines	2.7 ± 0.1	1.0 ± 0.1
2	‖ LIPSS	12 ± 2	0. 96 ± 0.05
	⊥ LIPSS	6.59 ± 0.06	0.6 ± 0.1
a-Si:H	–	0.018 ± 0.001	3.7 ± 0.1

## Data Availability

Data sharing is not applicable to this article.
